# Integrated Transcriptomic and Metabolic Analyses Highlight Key Pathways Involved in the Somatic Embryogenesis of *Picea mongolica*

**DOI:** 10.3390/plants14142141

**Published:** 2025-07-11

**Authors:** Jinling Dai, Shengli Zhang, Yu’e Bai

**Affiliations:** College of Forestry, Inner Mongolia Agricultural University, Hohhot 010019, China; daijinling2022@imau.edu.cn (J.D.); zhangshengli@imau.edu.cn (S.Z.)

**Keywords:** *Picea mongolica*, somatic embryogenesis, transcriptome, metabolome

## Abstract

In the severe environment of Hunshandake Sandy Land, the uncommon and indigenous Chinese tree species *Picea mongolica* is an important biological component. Conventional seed propagation in *P. mongolica* is constrained by low germination rates, prolonged breeding cycles, and hybrid offspring genetic instability, limiting efficient varietal improvement. In contrast, somatic embryogenesis (SE) offers superior propagation efficiency, exceptional germination synchrony, and strict genetic fidelity, enabling rapid mass production of elite regenerants. However, SE in *P. mongolica* is hampered by severe genotype dependence, poor mature embryo induction rates, and loss of embryogenic potential during long-term cultures, restricting the production of high-quality seedlings. In this study, we aimed to analyze the transcriptome and metabolome of three crucial phases of SE: mature somatic embryos (MSEs), globular somatic embryos (GSEs), and embryogenic calli (EC). Numerous differentially expressed genes (DEGs) were found, especially in pathways linked to ribosomal functions, flavonoid biosynthesis, and the metabolism of starch and sucrose. Additionally, 141 differentially accumulated metabolites (DAMs) belonging to flavonoids, organic acids, carbohydrates, lipids, amino acids, and other metabolites were identified. An integrated study of metabolomic and transcriptome data indicated considerable enrichment of DEGs and DAMs in starch and sucrose metabolism, as well as phenylpropanoid biosynthesis pathways, all of which are required for somatic embryo start and development. This study revealed a number of metabolites and genes linked with SE, offering important insights into the molecular mechanisms driving SE in *P. mongolica* and laying the groundwork for the development of an efficient SE system.

## 1. Introduction

*Picea mongolica* is a rare and indigenous tree species in China [1,2]. It is resistant to cold, drought, and barren conditions, forming unique forests in the harsh environment of the Hunshandake Sand Land system. The presence of *P. mongolica* effectively blocks the connection of sandy land, providing a solid foundation for constructing ecological security barriers in northern China. Because of its hardwood qualities and exquisite texture, *P. mongolica* is also a commercially significant tree species that is frequently used in furniture and building [1,3]. However, because of its low natural regeneration rate, late spring cold affecting seed production, and the influence of insect pests, the species resources and distribution range of *P. mongolica* have steadily declined, placing the species at risk of extinction [4].

Somatic embryogenesis (SE) holds both fundamental research and applied value, offering irreplaceable advantages in rapid propagation, genetic improvement, and germplasm conservation [5,6]. SE technique has found widespread application in artificial seed production, germplasm conservation, and molecular and cell engineering breeding [7,8,9]. Since Hakman et al. first reported successfully inducing SE and plant regeneration in *Picea abies* in 1985, SE has been successfully performed in more than 10 spruce species [10,11,12,13,14]. Plants regenerated through SE have been applied to silvicultural practices, yielding significant ecological and economic benefits [15]. Yan et al. used *P. mongolica* immature zygotic embryos as explants to create and optimize a SE system [16]. Furthermore, the transcriptomes of *P. mongolica*’s non-embryogenic calli (NEC) and embryogenic calli (EC) were sequenced by Wang et al. Differentially expressed genes (DEGs) were predominantly enriched in pathways associated with stress, signal transduction, and plant hormones, indicating a substantial difference in gene expression patterns between the two calli types [17].

Transcriptomic analysis was performed to elucidate the molecular mechanisms underlying SE in *Picea balfouriana*. Comparative transcriptome profiling between EC and NEC revealed significant alterations in genes associated with plant hormone signal transduction, starch and sucrose metabolism, and phenylpropanoid biosynthesis [18]. Furthermore, in genetically identical *P. abies* samples subjected to somatic embryo induction under varying temperatures, transcriptomic data identified DEGs involved in epigenetic regulation, histone methylation, temperature sensing, and signal transduction pathways [19,20]. In recent years, “multi-omics” methods, such as metabolomics, proteomics, genomics, and transcriptomics, have provided new insights into the mechanisms driving plant development and growth [21]. Integrating transcriptome and metabolome data enables the prediction of gene functions within a specific metabolic pathway while also offering additional evidence for gene identification. A combined transcriptome and metabolomic group analysis has been used to explore the molecular mechanism of SE in species such as *Camellia sinensis*, *Gossypium hirsutum*, and *Paeonia ostii* [22,23,24].

In conifers, *Larix kaempferi* and *Pinus koraiensis* cell lines with varying somatic embryogenic capacity were subjected to an integrated metabolomics and transcriptomics investigation. Numerous DEGs and DEMs were identified between cell lines with different somatic embryogenic capacities, primarily involved in plant signal transduction, starch and sugar metabolism, phenylpropane metabolism, and flavonoid metabolism. The expression levels of many totipotency-related transcription factor genes in EC are higher than those in NEC, which results in activating the expression of genes related to embryonic development [25,26]. Integrated multi-omics analyses (lipidomics, transcriptomics, proteomics) of dehydrated *Picea asperata* somatic embryos revealed molecular mechanisms of desiccation-induced lipid remodeling [27]. Comparative transcriptomic and metabolomic analyses of normal and aberrant *P. abies* somatic germinating embryos. The aberrant germinating embryos demonstrated significant sugar accumulation, with downregulated glucose signaling genes and upregulated starch biosynthesis genes, indicating crucial roles of sugar metabolic reprogramming during late embryogenesis and germination [28].

This study aimed to investigate the dynamic transcriptomic and metabolomic profiles of EC, globular somatic embryos (GSEs), and mature somatic embryos (MSEs) during SE in *P. mongolica*. We analyzed key metabolites and their associated DEGs, elucidating molecular and biochemical mechanisms underlying SE. Our findings enhance our understanding of conifer SE and may contribute to improving SE efficiency in *P. mongolica*.

## 2. Results

### 2.1. Induction of SE

**Figure 1:**
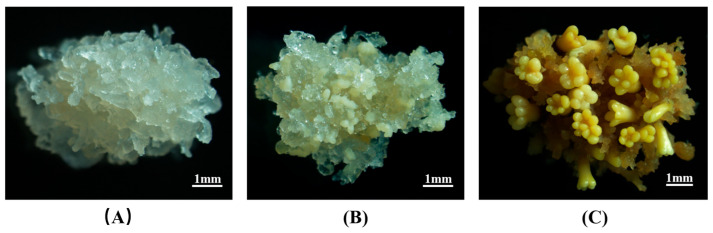
Different stages of *P. mongolica* SE. (**A**) Embryogenic calli (EC). (**B**) Globular somatic embryos (GSEs). (**C**) Mature somatic embryos (MSEs). Scale bars = 1 mm.

An efficient *P. mongolica* SE system was established, and cultures were obtained from different developmental stages, including EC, GSEs, and MSEs with cotyledonary structures, following our previously published method [16] (Figure 1). Samples from the three stages were collected for subsequent experiments.

### 2.2. Transcriptome Analysis of EC, GSEs, and MSEs

**Figure 2:**
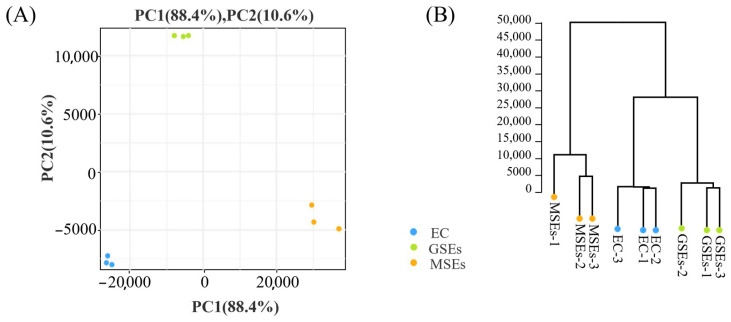
Principal component analysis (PCA) and hierarchical clustering plots. (**A**) PCA score plots. (**B**) Hierarchical clustering plots. Each point represents an independent biological replicate.

RNA-seq analysis of EC, GSEs, and MSEs produced 417,785,542 raw reads, 415,595,670 clean reads, and 61,330,034,039 bp of clean data. Q20 and Q30 averages were 97.93% and 93.66%, respectively. The average GC content was 44.40% (Table 1). Principal component analysis (PCA) demonstrated that there was a distinct demarcation between the groups, and the three biological replicates of the groups were grouped tightly together (Figure 2A). The results of hierarchical clustering were the same as those of PCA (Figure 2B). Overall, the samples’ depth and quality of transcriptome sequencing satisfied the requirements for further analysis.

#### 2.2.1. DEG Identification and Enrichment Analysis

**Figure 3:**
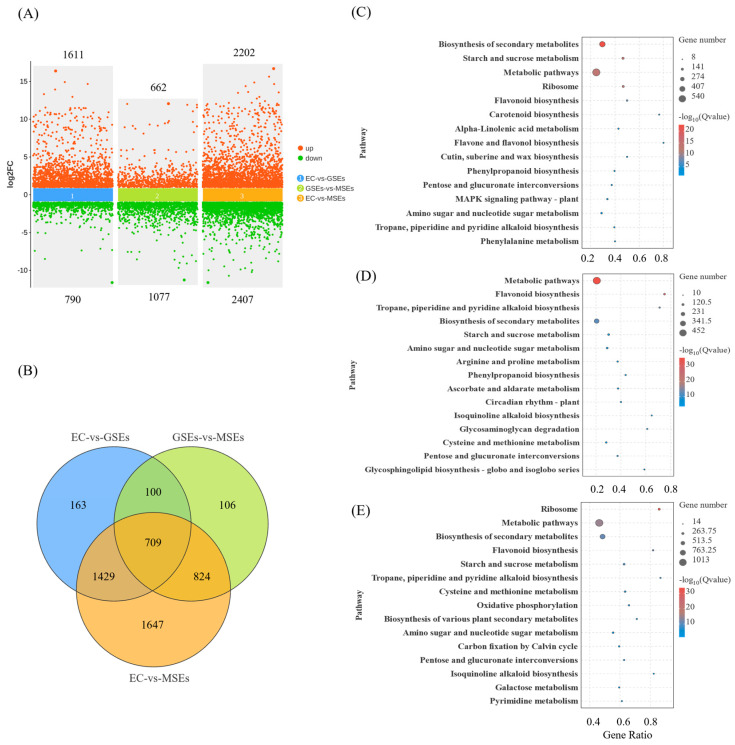
Transcriptomic analysis of EC, GSEs, and MSEs. (**A**) Scatter map of differentially expressed genes (DEGs) between the different comparison groups. (**B**) Venn diagram showing the DEGs for the various comparison groups. (**C**–**E**) DEGs enriched in different Kyoto Encyclopedia of Genes and Genomes (KEGG) pathways.

DEGs were defined as genes with an absolute fold change ≥ 2 and a false discovery rate < 0.05. The three developmental stages yielded a total of 4978 DEGs. A total of 790 and 1611 genes were downregulated and upregulated, respectively, in the EC vs. GSE comparison. In the GSE vs. MSE comparison, 1077 and 662 genes were downregulated and upregulated, respectively. In the EC vs. MSE comparison, 2407 and 2202 genes were downregulated and upregulated, respectively. A significant number of genes altered their patterns of expression in MSEs compared to EC (Figure 3A). The Venn diagram of the three datasets showed that the groups shared 709 DEGs (Figure 3B).

To better understand the biological functions of DEGs, a Gene Ontology (GO) enrichment analysis was conducted. The DEGs were enrichment in 3 cellular components, 17 chemical activities, and 26 biological processes. The DEGs in the EC vs. GSE and EC vs. MSE comparison groups were significantly increased in glycogen metabolism, starch biosynthesis, energy reserve metabolism, and amyloplasts. However, when comparing GSEs with MSEs, the DEGs associated with oxidoreductase, hydrolase, glycosyl bond, and catalytic activities were downregulated (Figure S1).

Kyoto Encyclopedia of Genes and Genomes (KEGG) enrichment analysis was utilized to assess the biological functions of the DEGs. The DEGs in the EC vs. GSE, GSE vs. MSE, and EC vs. MSE comparison groups were annotated to 114, 109, and 127 KEGG metabolic pathways, respectively. In the EC vs. GSE comparison, 17 metabolic pathways were significantly enriched, including starch and sucrose metabolism, α-linolenic acid metabolism, ribosome biosynthesis, flavonoid biosynthesis, and carotenoid biosynthesis (Figure 3C). In the GSE vs. MSE comparison, 30 metabolic pathways were significantly enriched, including starch and sucrose metabolism; tropane, piperidine, and pyridine alkaloid biosynthesis; and flavonoid biosynthesis (Figure 3D). In the EC vs. MSE comparison, 13 metabolic pathways were significantly enriched, including starch and sucrose metabolism; flavonoid and ribosome biosynthesis; and tropane, piperidine, and pyridine alkaloid biosynthesis (Figure 3E).

#### 2.2.2. Transcription Factor Expression Among EC, GSEs, and MSEs

**Figure 4:**
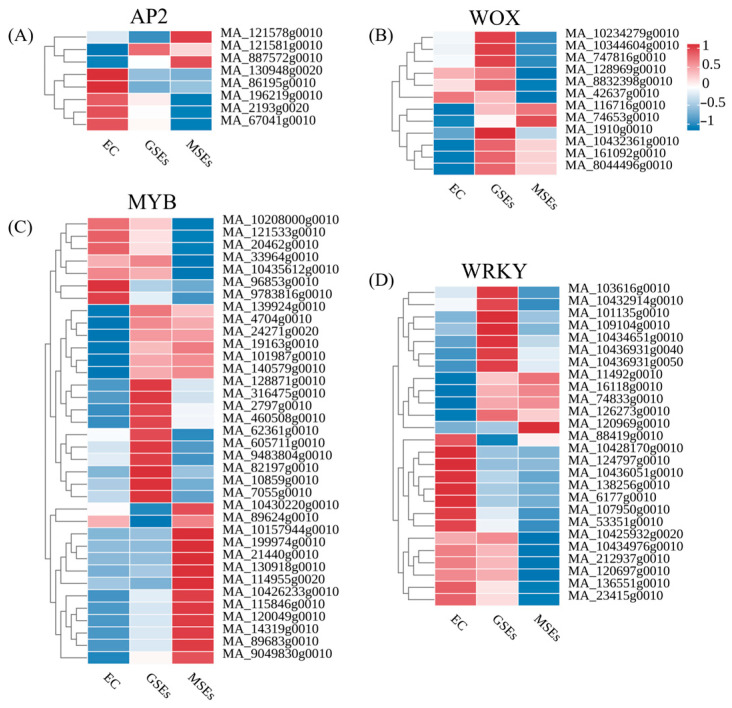
Differences in the expression levels of the AP2, WOX, MYB, and WRKY families of genes in EC, GSEs, and MSEs. (**A**) The heatmaps of DEGs related to AP2. (**B**) The heatmaps of DEGs related to WOX. (**C**) The heatmaps of DEGs related to MYB. (**D**) The heatmaps of DEGs related to WRKY.

Previous studies have established that transcription factor families, including AP2, WOX, WRKY, and MYB, play crucial regulatory roles in SE. Therefore, the expression levels of DEGs in these families were analyzed. A total of five AP2 genes were most highly expressed in EC; one was most highly expressed in GSEs, and two were most highly expressed in MSES (Figure 4A). WOX family members exhibited distinct expression profiles: one gene in EC, nine in globular GECs, and two in MSEs (Figure 4B). The MYB family demonstrated particularly dynamic regulation, with 6, 14, and 16 genes showing the highest expression in EC, GEC, and MSEs, respectively (Figure 4C). WRKY transcription factors followed this trend, with 13, 9, and 4 genes being most highly expressed in EC, GECs, and MSEs (Figure 4D).

#### 2.2.3. Verification of DEGs via qRT-PCR

**Figure 5:**
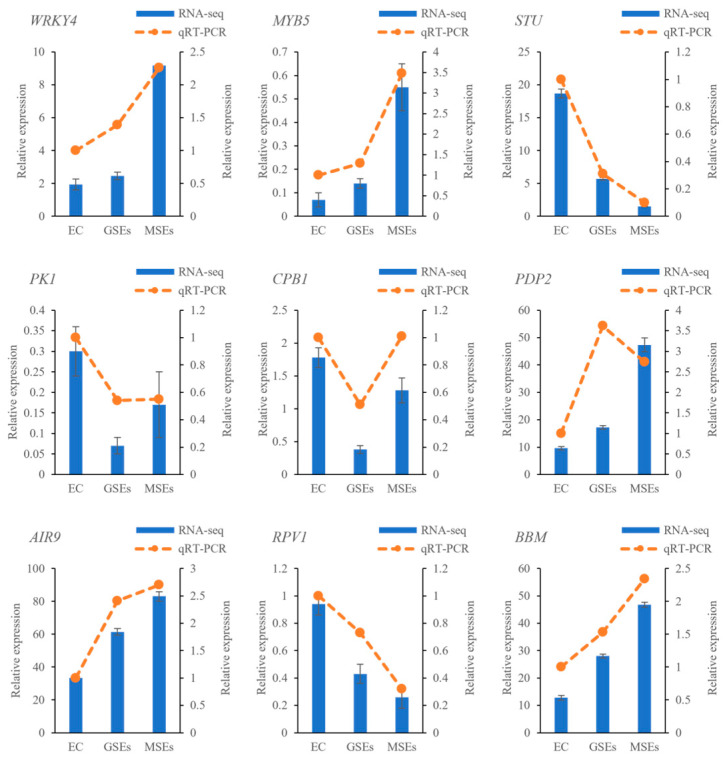
qRT-PCR examination of the selected DEGs (*WRKY4*, *MYB5*, *STU*, *PK1*, *CPB1*, *PDP2*, *AIR9*, *RPV1*, and *BBM*) identified in EC, GSEs, and MSEs.

To validate the transcriptome results, nine DEGs were evaluated using qRT-PCR, with the *EF1-α* gene serving as the internal reference gene. The comparison of transcriptome sequencing and qRT-PCR results on gene expression levels is shown in Figure 5. *WRKY*, *MYB5*, *AIR9*, and *BBM* were upregulated in GSEs and MSEs, whereas *STU*, *CBP1*, *PK1*, and *RPV1* were downregulated in GSEs. The transcriptome results were largely reliable, as eight out of nine genes showed consistent expression patterns across both methods, with the exception of *PDP2*.

### 2.3. Metabolomic Analysis of EC, GSEs, and MSEs

**Figure 6:**
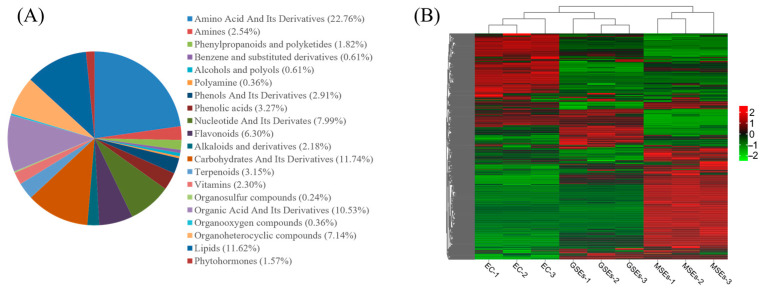
Metabolomic analysis of EC, GSEs, and MSEs. (**A**) EC, GSE, and MSE metabolite classification. (**B**) Heatmap of metabolite abundance of EC, GSEs, and MSEs.

Material from three developmental phases was submitted to broadly focused metabolomic analysis using LC-MS/MS, showing 826 metabolites spanning 20 categories (Table S1). Among these categories, amino acids and derivatives, carbohydrates, lipids, organic acids, and flavonoids represented the predominant metabolite groups (Figure 6A). PCA demonstrated strong clustering of biological replicates within each sample group, confirming high reliability and repeatability of the metabolomic data (Figure S2). Hierarchical clustering further validated the experimental consistency while highlighting significant metabolic differences among EC, GSEs, and MSEs (Figure 6B).

**Figure 7:**
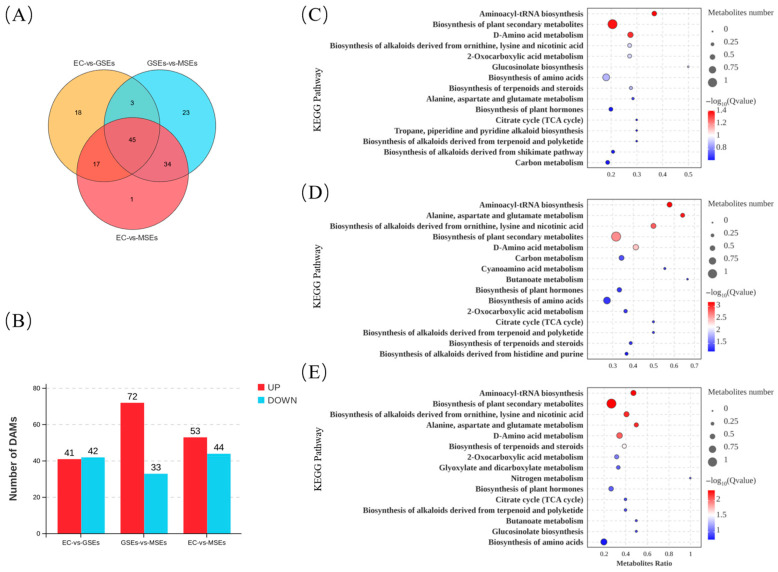
Statistical analysis of DAMs. (**A**) Venn diagram of DAMs across different comparison groups. (**B**) The quantity of DAMs in each comparative group. (**C**–**E**) DAMs enriched in various KEGG pathways.

A total of 141 DAMs belonging to broad classes of carbohydrates, amino acids, organic acids, flavonoids, lipids, and various other metabolites were identified in EC, GSEs, and MSEs (Table S2). Among these, there were 45 DAMs in EC, GSEs, and MSEs, with 18 DAMs unique to EC and GSEs and 23 DAMs unique to GSEs and MSEs. Oleamide was the sole DAM specific to EC and MSEs (Figure 7A). In addition, 83 DAMs (41 upregulated and 42 downregulated) were discovered in the EC vs. GSE comparison, whereas 105 DAMs (72 upregulated and 33 downregulated) were discovered in the GSE vs. MSE comparison. The EC vs. MSE analysis revealed 97 DAMs (53 upregulated and 44 downregulated) (Figure 7B).

The DAMs in the EC vs. GSE, GSE vs. MSE, and EC vs. MSE comparisons were assigned to 63, 75, and 76 pathways, respectively, according to KEGG enrichment analysis. There were 10 significantly enriched pathways in the EC vs. GSE comparison, including aminoacyl-tRNA and plant hormone biosynthesis; alanine, aspartate, and glutamate metabolism; and glucosinolate biosynthesis (Figure 7C). In contrast, there were 24 significantly enriched pathways in the GSE vs. MSE comparison. In addition to the biosynthesis of plant hormones and amino acids, metabolic pathways associated with alkaloid, terpenoid, and phenylpropanoid biosynthesis were significantly enriched (Figure 7D). The DAM enrichment results for the EC vs. MSE comparison group were similar to those of the EC vs. GSE comparison group, with 17 significantly enriched pathways (Figure 7E).

### 2.4. DEG and DAM Expression Trend Analysis

**Figure 8:**
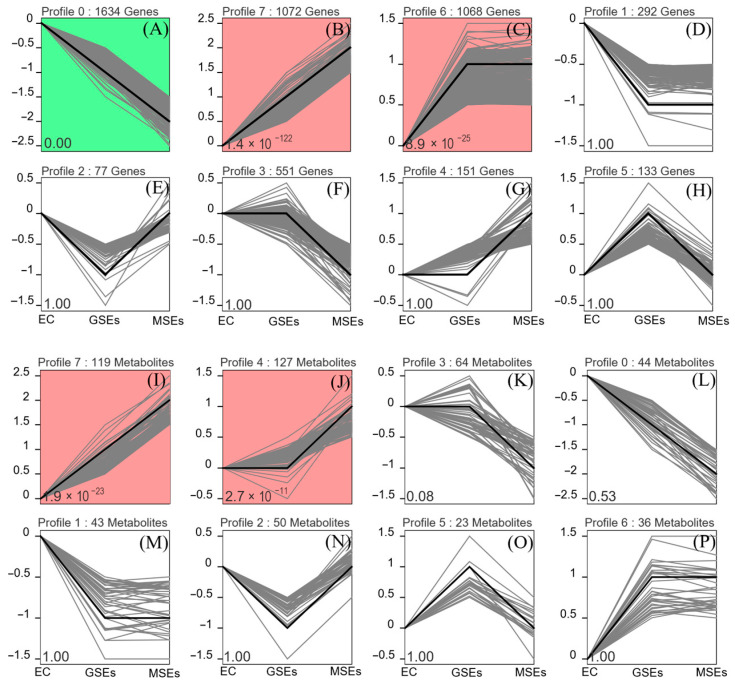
Series test of DEG and DAM clusters. (**A**–**H**) Different trends of DEGs expression. Each line represents a gene. (**I**–**P**) Different trends of DAM accumulation. Each line represents one metabolite. Colored trend clusters indicate statistically significant patterns (*p* < 0.05).

The DEGs and DAMs showed different expression trends during the EC, GSE, and MSE stages. As illustrated in Figure 7, trends are represented by color, with the same color indicating a shared pattern of enrichment. DEGs were significantly enriched in three trends: continuous decline (1634 genes) (Figure 8A), continuous rise (1072 genes) (Figure 8B), and rise followed by stabilization (1068 genes) (Figure 8C). There were two trends of significant DAM enrichment, both of which were upward, with 246 metabolites (Figure 8I,J). DAMs that were significantly increased included amino acids, carbohydrates, organic acids, and their derivatives, such as ergothioneine, nicotinic acid-hexoside, and hydroxycitric acid. In addition, two plant hormones, salicylic acid O-glucoside and trans-zeatin riboside, continued to accumulate during SE (Table S3). For the rising trend of DAMs and DEGs, KEGG enrichment analysis was performed (Figure S3). Enrichment results showed that phenylpropanoid and flavonoid biosynthesis, along with starch and sucrose biosynthesis, were the frequently enriched pathways. These results were similar to those obtained from the combined transcriptome and metabolome enrichment analysis.

### 2.5. Analysis of the Combined Transcriptome and Metabolome Data

Pathways enriched with both DEGs and DAMs were further analyzed. These molecules were predominantly concentrated in starch and sucrose metabolism, phenylalanine metabolism, carbon metabolism, amino acid biosynthesis, and ABC transporter pathways (Figure S4).

**Figure 9:**
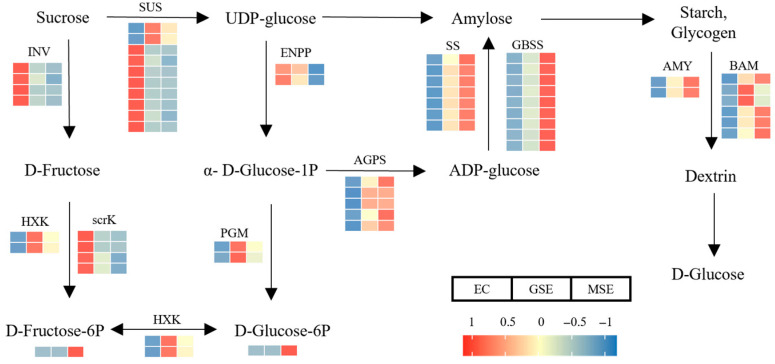
DAMs and DEGs involved in starch and sucrose metabolism pathways. Variations in DAM and DEG levels are depicted in the heatmaps. Enzyme abbreviations: SUS, sucrose synthase; INV, beta-fructofuranosidase; SS, starch synthase; ENPP, ectonucleotide pyrophosphatase; BAM, beta-amylase; AMY, alpha-amylase; PGM, phosphoglucomutase; GBSS, granule-bound starch synthase; HXK, hexokinase; scrK, sucrose kinase; AGPS, glucose-1-phosphate adenylyltransferase.

D-fructose-6-p and D-glucose-6-p were highly accumulated in MSEs in the starch and sucrose metabolic pathways (Figure 9), whereas other metabolites did not differ significantly at different stages of SE. The genes associated with β-fructofuranosidase, sucrose synthase, and fructokinase were strongly expressed in EC, whereas that of hexokinase was primarily expressed in GSEs. Granule-bound starch synthase and α-amylase exhibited elevated expression in MSEs. Starch and sucrose metabolism play key roles in cell division, tissue differentiation, and organogenesis. The high accumulation of fructose and glucose reflects the need for high carbohydrate and energy metabolism during SE.

**Figure 10:**
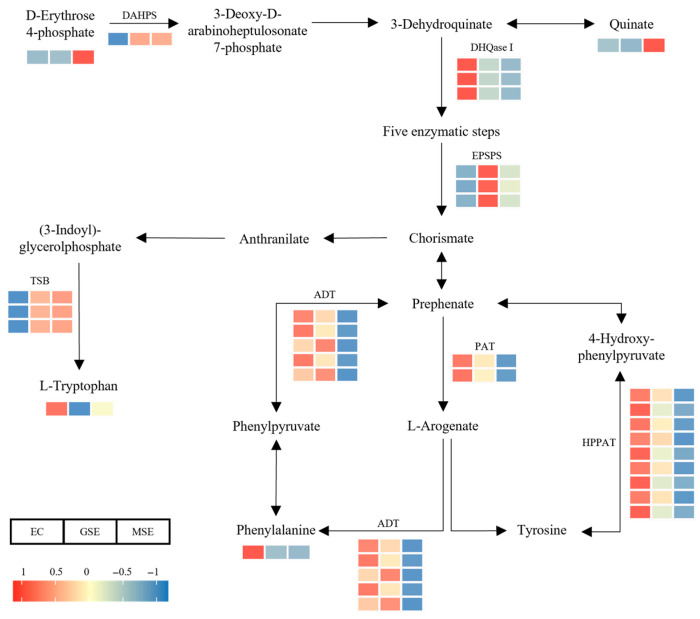
DAMs and DEGs involved in the phenylalanine, tyrosine, and tryptophan biosynthesis pathways. Variations in DAM and DEG levels are depicted in the heatmaps. Enzyme abbreviations: ADT, arogenate dehydratase; DAPHS, 3-deoxy-d-arabino-heptulosonate-7-phosphate synthase; DHQase I, 3-dehydroquinate dehydratase I; PAT, prephenate aminotransferase; EPSPS, 5-enolpyruvylshikimate-3-phosphate synthase; HPPAT, 4-hydroxyphenylpyruvate aminotransferase; TSB, tryptophan synthase beta chain.

A considerable number of the DAMs and DEGs participated in metabolic pathways linked to amino acid production. In the phenylalanine, tyrosine, and tryptophan biosynthesis pathways (Figure 10), L-tryptophan levels rose significantly in EC, decreased in GSEs, and then increased again in MSEs. Phenylalanine was significantly enriched only in EC, while D-erythrose 4-phosphate and quinate accumulated substantially in MSEs. Genes involved in synthesizing these amino acids, such as prephenate dehydratase and aspartate aminotransferase (involved in phenylalanine biosynthesis), exhibited similar expression patterns. The expression of tryptophan synthase alpha chain also increased throughout SE.

## 3. Discussion

The regulatory network governing SE is highly complex, particularly in woody plants, where the mechanistic underpinnings remain largely unresolved [29,30]. In contrast to model organisms like *Arabidopsis thaliana*, research on SE in woody plants is comparatively limited, posing significant challenges to the comprehensive elucidation of its regulatory framework [31]. This study aimed to systematically delineate the potential regulatory network underlying SE in *P. mongolica* through the integrated metabolomic and transcriptomic profiling of EC, GSEs, and MSEs. The findings revealed that SE in *P. mongolica* is regulated by multiple metabolic pathways, including starch and sucrose metabolism, phenylpropanoid metabolism, amino acid metabolism, and hormone signaling cascades.

### 3.1. Role of Starch and Sucrose Metabolism in SE

Plant cell division, tissue differentiation, and organ formation are all significantly impacted by starch and sucrose metabolism [32]. Compared to GSEs and MSEs, EC exhibited a substantially greater sucrose content in this study. Furthermore, genes, such as α-amylase (*AMY*), β-amylase (*BMY*), granule-bound starch synthase (*GBSS*), and starch synthase (*SS*), were markedly upregulated in GSEs and MSEs. These findings align with previous studies showing elevated starch content in EC versus NEC, accompanied by numerous DEGs and DAMs in the starch and sucrose metabolism pathways [25]. Collectively, these results underscore the critical involvement of starch and sucrose metabolism in SE, where they facilitate proper embryonic development through energy provision and metabolic regulation.

Additionally, sucrose acts as a signaling molecule that regulates the expression of genes related to primary and secondary metabolism, thereby influencing cellular development [33]. Also, the expression of key flavonoid biosynthesis genes (e.g., *F3′H*, *F3H*, *C4H*, and *CHI*) correlates with sucrose concentration in the medium. This correlation was reported in other species too: increasing sucrose levels in grape callus cultures significantly enhance flavonoid production and cell proliferation [34].

### 3.2. Role of Amino Acid and Flavonoid Metabolism in SE

Amino acids act as metabolic precursors, signaling molecules, and antioxidants, playing crucial regulatory roles throughout various stages of plant SE [35]. This study identified numerous DEGs and DAMs closely associated with amino acid biosynthesis. Significant accumulations of amino acids, including glutamine, tryptophan, and phenylalanine, were observed in the EC stage, which declined in the GSE stage and increased in the MSE stage. Similar trends have been reported in the SE of tea and litchi [36,37]. In plants, phenylalanine and tryptophan are key precursors of indole-3-acetic acid (IAA). IAA is necessary for controlling the cell cycle, differentiation, and organ creation during SE, which are required for frequent cell division and differentiation [38].

Phenylalanine is the initial substrate of the phenylalanine pathway and is converted into 4-coumaryl-CoA to synthesize different flavonoid compounds [39]. Numerous DEGs in the flavonoid and phenylpropanoid biosynthesis pathways were significantly upregulated during SE in *P. mongolica*. For instance, metabolomic analyses of GSEs and EC in cotton have revealed that flavonoids are characteristic metabolites that accumulate during somatic embryo development. Integrated transcriptomic and metabolomic analyses further demonstrated the significant accumulation of metabolites in the flavone, flavonol, flavonoid, and phenylpropanoid biosynthesis pathways in both EC and GSEs [23]. Similarly, metabolomic profiling of SE-potential cell lines in Korean pine demonstrated that compounds like caffeic acid, coumaric acid, and phenylalanine were significantly more abundant in high-potential lines than in lines with low differentiation potential [26]. These findings suggest that phenylalanine metabolism and flavonoid compound synthesis may play pivotal regulatory roles in the SE of *P. mongolica*.

### 3.3. Plant Hormone Signal Transduction in SE

Previous reports have indicated that hormone signal transduction activates a variety of developmental pathways, and their interplay regulates SE induction [40,41]. Although the study identified DEGs in the hormone signaling transduction pathway, it was not analyzed in detail because the pathway was not significantly enriched. During SE, the IAA receptor Transport Inhibitor Response 1 (TIR1) was markedly upregulated. TIR1 binds to AUX/IAA proteins, triggering their degradation and releasing auxin response factor (ARF) transcription factors, which then regulate genes involved in embryogenesis. For example, GH3 exhibited an expression pattern of initial downregulation, followed by upregulation during SE. Studies have suggested that low IAA concentrations are necessary to promote cell differentiation and somatic embryo formation. The importance of IAA has been demonstrated in SE studies on coffee and peony [24,42].

During somatic embryo maturation in *P. mongolica*, a high concentration of abscisic acid (ABA) was applied. ABA functions by binding to its receptors, pyrabactin resistance/PYR1-Like (PYR/PYL), which inhibit protein phosphatase 2C (PP2C). This inhibition activates SNF1-related protein kinase 2 (SnRK2), subsequently regulating genes involved in embryo maturation. The expression levels of PYR/PYL, PP2C, and SnRK2 were significantly upregulated. Similarly, ABA levels notably increased during SE in two rubber tree cell line genotypes. In a study on the SE of *Qercus aliena*, appropriate concentrations of ABA effectively promoted SE [43].

Furthermore, this study found that the CORONATINE INSENSITIVE 1 (*COI1*) gene was significantly upregulated during SE, promoting the degradation of JASMONATE ZIM-domain (JAZ) proteins and the release of transcription factors, such as MYC2, which regulate embryo development and morphogenesis. However, the specific mechanisms of action of JA in SE, particularly its regulatory network involving transcription factors and downstream genes, remain to be elucidated. Future research should focus on modulating the JA signaling pathway to improve SE efficiency and quality.

This study had some limitations. First, the static sampling design, which focused on three developmental stages (EC, GSEs, and MSEs), may not accurately capture the dynamic transcriptional and metabolic alterations that occur during SE. Second, the limited sensitivity of metabolomics in detecting low-abundance metabolites compared to the ability of transcriptomics to detect low-expression genes may result in data imbalance. Third, the observed correlations between gene expression and metabolite levels likely reflect indirect associations mediated by multi-level regulatory mechanisms rather than direct causal relationships. Future studies incorporating time-series sampling, advanced instrumentation technology, and proteomic data may help address these limitations and provide more information regarding the molecular mechanisms underlying SE.

## 4. Materials and Methods

### 4.1. Plant Material

Immature seeds of *P. mongolica* were harvested in July 2023 from Hohhot, China. The methods and medium for inducing somatic embryos were described by Yan et al. [15]. Representative samples of EC, GSEs, and MSEs were collected, immediately frozen in liquid nitrogen, and stored at −80 °C for subsequent transcriptomic and metabolic analyses.

### 4.2. Transcriptomic Analysis

Using a TRIzol reagent kit (Invitrogen, Carlsbad, CA, USA), total RNA was isolated from EC, GSEs, and MSEs. The cDNA library was built according to the Illumina guidelines. Sequencing was performed using the Illumina HiSeqTM 4000 (San Diego, CA, USA). Fastp (version 0.18.0) was used to filter high-quality clean reads [44]. Using RNA-seq by expectation maximization (RSEM), the clean reads were mapped to the *P. abies* genome (https://plantgenie.org/ accessed on 3 January 2025) [45]. After normalizing the data to reads per kilobase per million reads, gene abundance was computed. R software (package gmodels v2.18.1, v4.3.0) (https://www.r-project.org/) was used to compute Pearson’s correlation coefficient and conduct PCA. DEGs were defined as genes that had an absolute fold change of at least 2 and a false discovery rate of less than 0.05. These genes were then further annotated and enriched in GO and KEGG [46].

### 4.3. Quantitative Real-Time PCR Analysis

Nine DEGs were selected for qRT-PCR to verify the RNA-seq results. Total RNA was extracted from EC, GSEs, and MSEs using the OminiPlant RNA Kit (CW2598S; ComWin, Beijing, China). RNA was reverse transcribed using the PrimeScript^TM^ RT Reagent Kit with gDNA Eraser (RR047A; TaKaRa, Dalian, China). Specific primers were designed using the Primer5.0 software (Table S4). PCR amplification was performed on a LightCycler 480 real-time PCR system (Roche, Basel, Switzerland) using TB Green Premix Ex Taq II (RR820B; Takara, Dalian, China). The PCR program was as follows: 45 cycles of 95 °C for 20 s, 58 °C for 30 s, and 72 °C for 30 s. Relative expression was calculated using the 2^−ΔΔCt^ method, with *EF1-α* serving as the internal control.

### 4.4. Metabolomic Analysis

The extraction and identification of metabolites from EC, GSE, and MSE samples were carried out using the standard procedures proposed by Gene Denovo (Guangzhou, China) [47]. DAMs were identified based on a *t*-test *p*-value < 0.05 and an OPLS-DA model variable importance in projection (VIP) > 1 [48,49]. Heatmap analysis was conducted using R. Functional annotation and enrichment analysis of DAMs were performed using the KEGG database [50]. Metabolites and genes with similar expression patterns in EC, GSE, and MSE samples were clustered. Each sample’s expression data were standardized and then clustered using the Short Time-series Expression Miner program (version 1.3.11) [51].

## 5. Conclusions

To investigate putative metabolic regulatory networks during SE in *P. mongolica*, we integrated transcriptomic and metabolomic analyses of EC, GSEs, and MSEs using RNA-seq and UPLC-MS/MS technologies. Enrichment analysis revealed the involvement of multiple metabolic pathways, including amino acid metabolism, starch and sucrose metabolism, phenylpropanoid biosynthesis, and plant hormone signal transduction, all of which play crucial roles throughout SE. Although sucrose levels decreased during SE, the production of fructose, glucose, and starch, alongside the upregulation of related genes, increased significantly to support the rapid cell division and differentiation required for SE. The upregulation of phenylpropanoid and flavonoid biosynthesis, using phenylalanine as a substrate, enhanced the antioxidant capacity, thereby promoting the initiation and development of SE. This comprehensive dataset provides valuable transcriptomic and metabolomic resources for *P. mongolica*, establishing a foundation for future research on its SE.

## Figures and Tables

**Table 1:** Overview of transcriptome sequencing of EC, GSEs, and MSEs.

Sample	Raw Reads	Clean Reads	Clean Data (bp)	Q20 (%)	Q30 (%)	GC Content (%)
EC-1	40,747,192	40,491,600	5,959,054,101	97.79	93.17	44.32
EC-2	57,552,526	57,200,990	8,401,685,909	98.05	94.26	44.32
EC-3	48,070,218	47,841,264	7,091,543,345	97.88	93.42	44.42
GSEs-1	40,351,730	40,153,750	5,959,722,664	97.96	93.69	44.60
GSEs-2	48,316,596	48,055,482	7,092,902,035	98.37	94.99	44.56
GSEs-3	45,805,840	45,538,794	6,677,507,898	97.73	93.00	44.64
MSEs-1	49,113,436	48,870,328	7,240,911,276	97.98	94.08	44.06
MSEs-2	45,370,116	45,170,886	6,683,159,328	97.86	93.37	44.30
MSEs-3	42,457,888	42,272,576	6,223,547,483	97.72	92.95	44.42
Summary/Average	417,785,542	415,595,670	61,330,034,039	97.93	93.66	44.40

## Data Availability

All sequence data were deposited in the NCBI Sequence Read Archive under the BioProject study PRJNA1236525 (Samples: SAMN47393702-SAMN47393710).

## Supplementary Materials

The following supporting information can be downloaded at: https://www.mdpi.com/article/10.3390/plants14142141/s1. Figure S1. GO enrichment analysis of DEGs; Figure S2. PCA score plots of transcriptome results. Figure S3. KEGG enrichment analysis of rising trend DAMs and DEGs. Figure S4. Heatmap of DEGs associated with the plant hormone signal transduction pathway. Table S1. Total number of metabolites detected in EC, GSEs, and MSEs. Table S2. Depiction of 141 DAMs in EC, GSEs, and MSEs. Table S3. Metabolites that accumulate continuously during SE. Table S4. qRT-PCR primer information.

## Abbreviations

The following abbreviations are used in this manuscript:

2,4-D	2,4-dichlorophenoxyacetic acid
6-BA	6-benzyladenine
ABA	Abscisic acid
ARF	Auxin response factor
BBM	Baby boom
DAMs	Differentially accumulated metabolites
DEGs	Differentially expressed genes
EC	Embryogenic calli
GO	Gene Ontology
GSEs	Globular somatic embryos
IAA	Indole-3-acetic acid
JA	Jasmonic acid
KEGG	Kyoto Encyclopedia of Genes and Genomes
LEC	Leafy cotyledon
MSEs	Mature somatic embryos
NEC	Non-embryogenic calli
PCA	Principal component analysis
SE	Somatic embryogenesis
WUS	Wuschel
