# Supplementary figures and images for: Integrated Transcriptomic and Metabolic Analyses Highlight Key Pathways Involved in the Somatic Embryogenesis of Picea mongolica
Source: Plants (Basel). 2025 Jul 11;14(14):2141. doi: 10.3390/plants14142141 (PMC12299269; doi:10.3390/plants14142141)

Figure S1

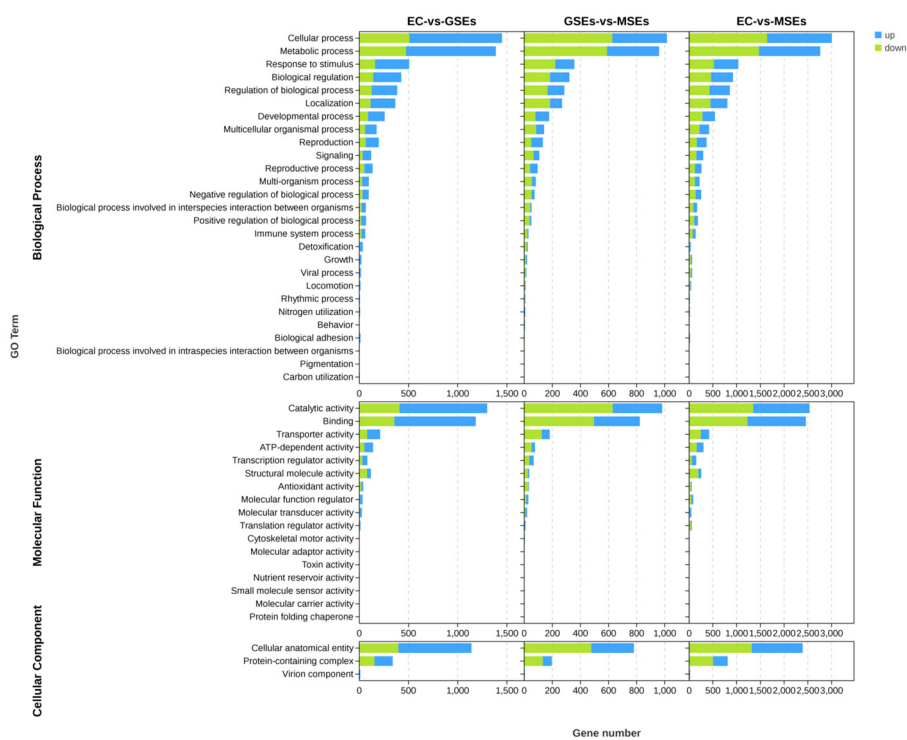

Figure S2

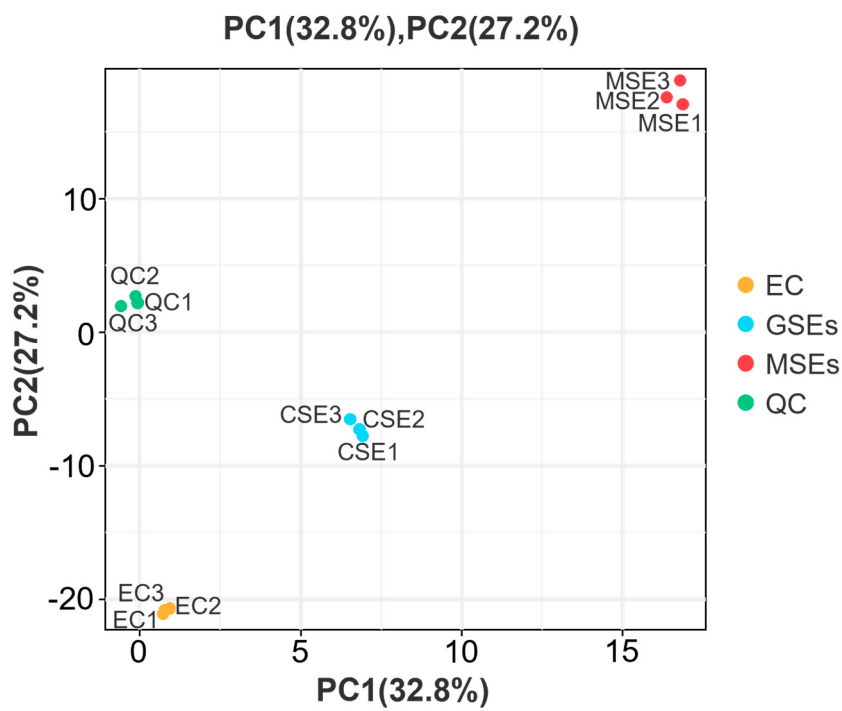

Figure S3

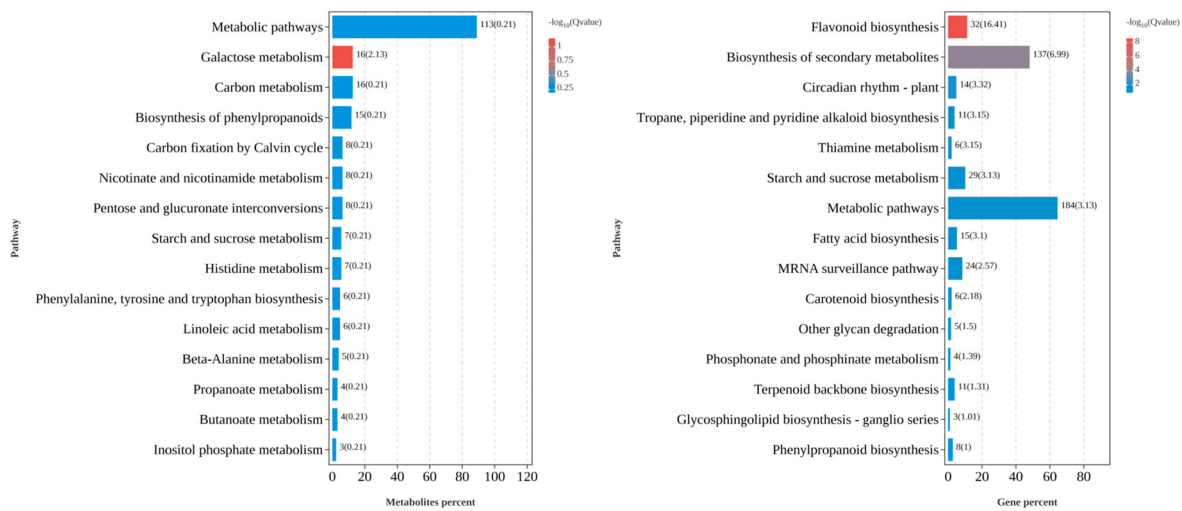

Figure S4

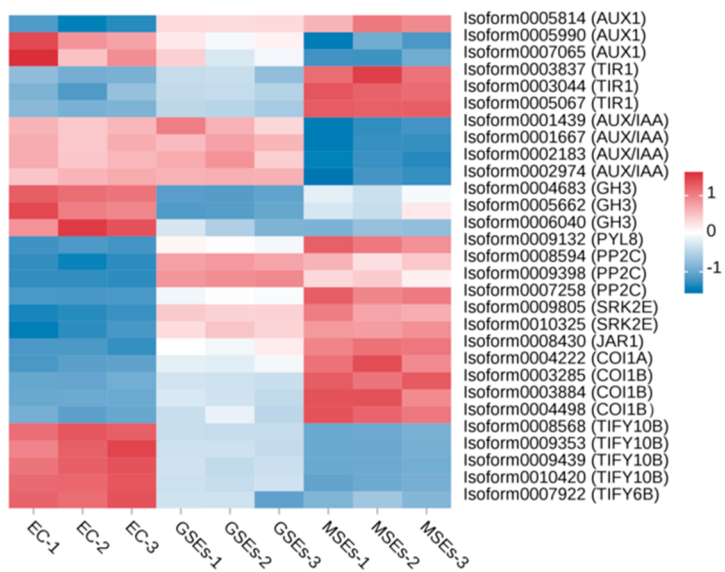

Supplement: Supplementary file 1 [file plants-14-02141-s001.zip › plants-3735037-supplementary figures.pdf]
